# Metagenome-assembled genomes of the GU0601 sample (the Han River, South Korea)

**DOI:** 10.1128/MRA.00688-23

**Published:** 2023-11-20

**Authors:** YeongJun Jang, Jae-Shin Kang, Eun Hee Bae, JunMo Lee

**Affiliations:** 1 Department of Oceanography, Kyungpook National University, Daegu, South Korea; 2 Biodiversity Research and Cooperation Division, National Institute of Biological Resources, Incheon, South Korea; 3 Climate Change and Environmental Biology Research Division, National Institute of Biological Resources, Incheon, South Korea; 4 Kyungpook Institute of Oceanography, Kyungpook National University, Daegu, South Korea; California State University San Marcos, San Marcos, California, USA

**Keywords:** metagenome-assembled genomes, cyanobacteria, proteobacteria, flavobacteria

## Abstract

We generated metagenome sequences of the GU0601 sample collected from the Han River and constructed metagenome-assembled genomes (MAGs) to identify their bacterial composition. We identified six MAGs belonging to Alphaproteobacteria, Cyanobacteria, and Flavobacteria.

## ANNOUNCEMENT

Freshwater environments are important habitats in nutrient cycling and food supply for diverse organisms and are necessary for industrial activities (1, 2). To study the bacterial community in the Han River, we collected the sample (GU0601) from the Han River, South Korea (37°34′23.3″N 127°08′02.5″E; 21 December 2020) and cultured it in CB medium (3) for the enrichment of the bacterial community (25°C; 12 h-light:12 h-dark; 3 weeks). The bacterial cells were collected by centrifugation (4°C, 30 min, 14,000 rpm; TOMY MX-307, TOMY, Japan), and these were ground using a mortar with liquid nitrogen for DNA extraction. The DNA extraction experiment was done by CTAB method (4). For the sequencing library preparation at PacBio, the genomic DNA molecules were sheared to 10–12 Kbp fragments during the size selection step using the Megaruptor v3 system (Diagenode, Belgium), and the DNA fragments were purified using AMPure PB bead purification system (0.45×; Pacific BioSciences, USA). For the sequencing library construction, we used SMRTbell Express Template Prep Kit 2.0 (Pacific BioSciences, USA). The genome sequencing was done by PacBio Sequel II SMRT HiFi with the manufacturer’s instructions. The HiFi sequencing reads of the GU0601 sample (11.4 Gbp; average read length = 9,153 bp; SRR25183167; PRJNA992221) were analyzed using the LongQC program (v1.2.0) with the “pb-hifi” option (5) and assembled by the hifiasm-meta assembler (v0.3-r063.2) with default options (6). The assembled sequences are 62.9 Mbp (1,422 contigs; N50 = 867,067 bp).

To predict metagenome-assembled genomes (MAGs), we analyzed our metagenome assembly using the MetaBAT2 program with default options (7). A total of 15 MAGs were initially predicted. To construct high-quality bacterial genomes, the contigs of each MAG were re-assembled using the *de novo* assembly function in Geneious Prime (v2023.1.2). We manually confirmed and accepted each re-assembled MAG that allowed for identical (or one nucleotide mismatched) overlapping between the re-assembled contigs. We predicted and annotated protein-coding sequences (CDSs), 16S rRNA, and tRNA of the MAGs by prokka program (v1.11) with default options (8). To validate the genome quality of the re-assembled MAGs, we performed Benchmarking Universal Single-Copy Orthologs (BUSCO; v5.4.4) analysis using “bacterial_odb10” with default options (9). To define high-quality MAGs, we used the following three criteria: (i) the longest contigs from each re-assembled MAG showed 95%–100% of BUSCO values, (ii) the number of gene contents in the MAGs was similar to that of their closely related reference bacterial genomes (the most three homologous genomes from mega BLASTn search; NT database), and (iii) the 16S-23S-5S rRNA contents were present in the MAGs. These criteria were referenced from the source (10). As a result, a total of six MAGs were defined as high-quality MAGs. The MAG3 and MAG4 were confirmed as circular bacterial genomes (Table 1) using Geneious Prime (v2023.1.2), which identifies circular genome structures based on identical sequences and their orientation at the ends of the contigs. We additionally confirmed the circular genome structures of the MAGs based on structural comparison with available reference bacterial genomes using the align function in Geneious Prime.

**TABLE 1:** MAGs of the GU0601 sample

MAGs	Genome size and BUSCO	GC (%)	Length of 16S rRNA sequences (MAGs) and mega BLASTn search top three (uncultured bacteria were excluded)	Gene contents	Available reference genomes (NCBI) from mega BLASTn search top 100
MAG1	5.2 Mbp, 100% BUSCO	68.0	# Predicted 16S rRNA length (MAG1): 1,484 bp *Bosea* sp. (CP016464; identity 100%; align length 1,484 bp) *Afipia* sp. (U87779; identity 100%; align length 1,457 bp) *Bosea* sp. (MW196647; identity 100%; align length 1,425 bp)	CDS 4,918, rRNA 6, tRNA 52	CP016464 (4.9 Mbp, CDS 4,632, rRNA 6, and tRNA 49) CP126307 (5.9 Mbp, CDS 5,771, rRNA 6, and tRNA 48) CP017147 (6.3 Mbp, CDS 5,860, rRNA 6, and tRNA 49)
MAG2	4.2 Mbp, 100% BUSCO	63.7	# Predicted 16S rRNA length (MAG2): 1,462 bp *Rhodobacter amnigenus* (MK404751, identity 97.89%; align length 1,391 bp) *Rhodobacter amnigenus* (NR_180404, identity 97.89%; align length 1,391 bp) *Tabrizicola* sp. (KP731978, identity 97.50%; align length 1,393 bp)	CDS 4,110, rRNA 6, tRNA 51	CP034328 (4.1 Mbp, CDS 4,003, rRNA 6, and tRNA 46) CP124535 (3.8 Mbp, CDS 3,670, rRNA 6, and tRNA 48) CP028918 (3.6 Mbp, CDS 3,525, rRNA 6, and tRNA 48)
MAG3 (circular)	5.1 Mbp, 95.9% BUSCO	40.5	# Predicted 16S rRNA length (MAG3): 1,490 bp *Oscillatioria* sp. (GQ351577; identity 99.39%; align length 1,457 bp) *Planktothrix paucivesiculata* (NR_177722; identity 99.11%; align length 1,454 bp) *Oscillatoria* sp. (GQ351574; identity 99.11%; align length 1,454 bp)	CDS 5,913, rRNA 11, tRNA 42	OW445656 (5.4 Mbp, CDS 4,328, rRNA 9, and tRNA 39) LO018304 (4.7 Mbp, CDS 4,328, rRNA 9, and tRNA 39) LR882952 (4.7 Mbp, CDS 4,275, rRNA 11, and tRNA 42)
MAG4 (circular)	3.1 Mbp, 100% BUSCO	65.4	# Predicted 16S rRNA length (MAG4): 1,481 bp *Porphyrobacter* sp. (LC094534; identity 100%; align length 1,370 bp) Alpha proteobacterium (LC217398; identity 99.79%; align length 1,425 bp) *Erythrobacter* sp. (CP117516; identity 99.73%; align length 1,477 bp)	CDS 2,911, rRNA 3, tRNA 44	CP117516 (3.6 Mbp, CDS 3,348, rRNA 3, and tRNA 44) CP017113 (2.9 Mbp, CDS 2,842, rRNA 3, and tRNA 47) CP041222 (3.7 Mbp, CDS 3,480, rRNA 3, and tRNA 46)
MAG5	2.7 Mbp, 100% BUSCO	32.1	# Predicted 16S rRNA length (MAG5): 1,510 bp *Flavobacterium* sp. (CP110008; identity 98.81%; align length 1,494 bp) *Flavobacterium* sp. (KM875710; identity 98.69%; align length 1,493 bp) *Flavobacterium* sp. (KU529208; identity 98.69%; align length 1,493 bp)	CDS 2,532, rRNA 9, tRNA 48	CP089095 (3.0 Mbp, CDS 2,670, rRNA 9, and tRNA 46) CP089096 (2.8 Mbp, CDS 2,558, rRNA 9, and tRNA 45) CP096205 (3.8 Mbp, CDS 3,282, rRNA 9, and tRNA 44)
MAG6	3.0 Mbp, 100% BUSCO	58.1	# Predicted 16S rRNA length (MAG6): 1,488 bp *Sphingopyxis* sp. (HQ436495; identity 100%; align length 1,425 bp) *Sphingomonas* sp. (AJ620195; identity 99.73%; align length 1,479 bp) *Sphingomonas* sp. (AJ620199; identity 99.66%; align length 1,478 bp)	CDS 2,895, rRNA 3, tRNA 45	CP035733 (3.4 Mbp, CDS 3,234, rRNA 3, and tRNA 43) CP018154 (2.5 Mbp, CDS 2,369, rRNA 6, and tRNA 41)

## Data Availability

Raw reads of genome sequencing data in this study were uploaded to the NCBI Sequence Read Archive (SRA) database (SRR25183167; Assembly GCA_030545275; BioSample SAMN36345608; BioProject PRJNA992221). The information regarding genome sequences and gene annotations of the MAGs in this study have been deposited at FigShare (https://doi.org/10.6084/m9.figshare.24278062).
